# Chronic activation of MUC1-C in wound repair promotes progression to cancer stem cells

**DOI:** 10.20517/2394-4722.2022.03

**Published:** 2022-03-31

**Authors:** Donald W. Kufe

**Affiliations:** Dana-Farber Cancer Institute, Harvard Medical School, Boston, MA 02115, USA.

**Keywords:** *MUC1*, MUC1-C, wound repair, CSC, lineage plasticity, epigenetic reprogramming, chromatin remodeling

## Abstract

The *mucin 1 (MUC1)* gene emerged in mammals to afford protection of barrier epithelial tissues from the external environment. *MUC1* encodes a transmembrane C-terminal (MUC1-C) subunit that is activated by loss of homeostasis and induces inflammatory, proliferative, and remodeling pathways associated with wound repair. As a consequence, chronic activation of MUC1-C promotes lineage plasticity, epigenetic reprogramming, and carcinogenesis. In driving cancer progression, MUC1-C is imported into the nucleus, where it induces NF-κB inflammatory signaling and the epithelial-mesenchymal transition (EMT). MUC1-C represses gene expression by activating (i) DNA methyltransferase 1 (DNMT1) and DNMT3b, (ii) Polycomb Repressive Complex 1 (PRC1) and PRC2, and (iii) the nucleosome remodeling and deacetylase (NuRD) complex. PRC1/2-mediated gene repression is counteracted by the SWI/SNF chromatin remodeling complexes. MUC1-C activates the SWI/SNF BAF and PBAF complexes in cancer stem cell (CSC) models with the induction of genome-wide differentially accessible regions and expressed genes. MUC1-C regulates chromatin accessibility of enhancer-like signatures in association with the induction of the Yamanaka pluripotency factors and recruitment of JUN and BAF, which promote increases in histone activation marks and opening of chromatin. These and other findings described in this review have uncovered a pivotal role for MUC1-C in integrating lineage plasticity and epigenetic reprogramming, which are transient in wound repair and sustained in promoting CSC progression.

## INTRODUCTION

Epithelial barrier tissues play a critical role in protecting mammals from insults, such as damage and infection, that arise from exposure to the external environment. Simple epithelia lining the respiratory, gastrointestinal, and genitourinary tracts are poised to respond to a loss of homeostasis by initiating inflammation and wound repair. More complex epithelial barriers, for example, in the skin, similarly respond to stress by activating inflammatory and repair signaling pathways^[1]^. These responses are driven by epithelial stem cells (SCs) that populate local niches and interact with the innate and adaptive immune microenvironment^[1]^. Resident SCs are dependent on lineage plasticity for maintaining homeostasis and promoting wound repair^[2]^.

**Figure 1.**
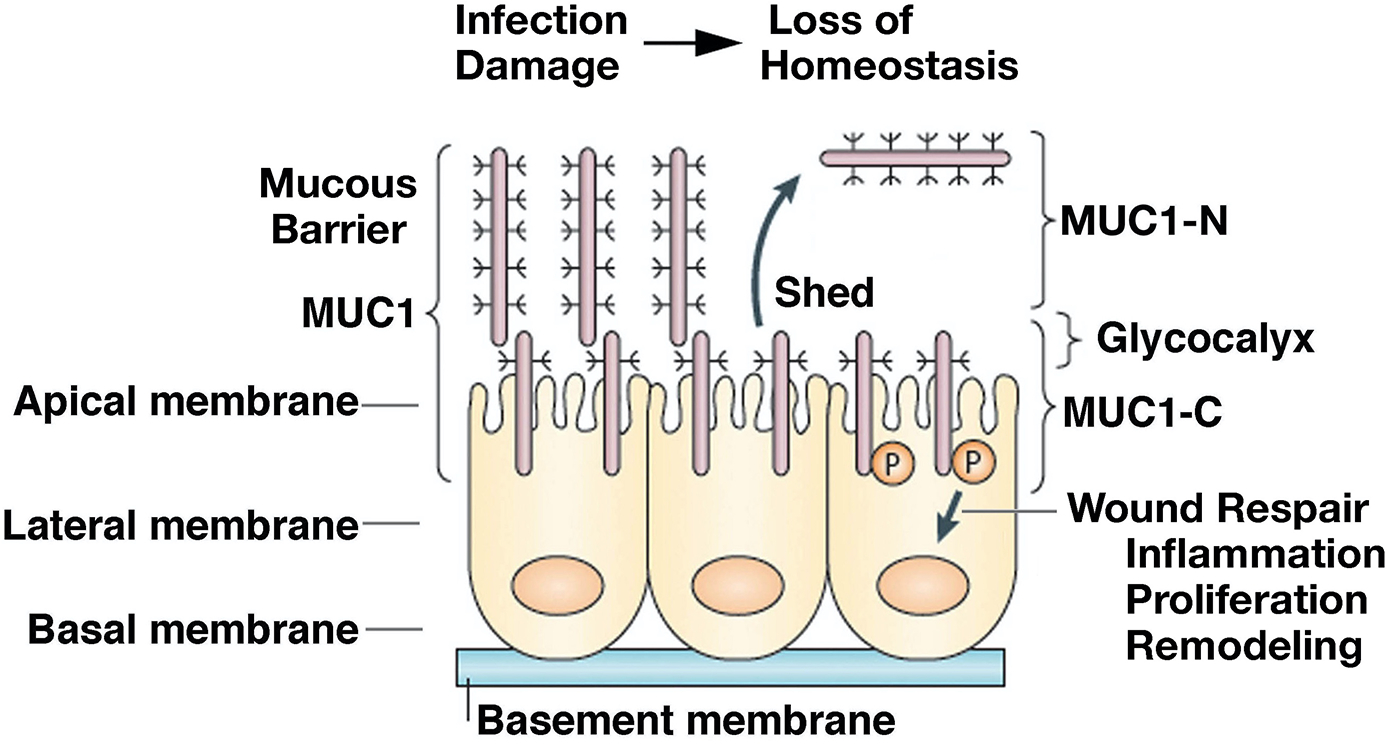
*MUC1* evolved to protect epithelia from the loss of homeostasis. *MUC1* encodes a single polypeptide that undergoes autocleavage into N-terminal (MUC1-N) and C-terminal (MUC1-C) subunits. MUC1-N and MUC1-C form a stable non-covalent complex mediated by a leucine zipper-like structure^[12]^. The MUC1-N/MUC1-C complex is positioned at the apical borders of polarized epithelial cells. In response to the loss of homeostasis associated with pathogenic infections, damage, and other forms of stress, entropic forces in the glycocalyx promote disruption of the MUC1-N/MUC1-C complex with shedding of MUC1-N into the protective mucous barrier. In turn, activation of MUC1-C induces inflammatory, proliferative, and remodeling signaling pathways associated with wound repair^[6,13]^. Figure modified from Kufe^[5,6]^. *MUC1*: The *mucin 1* gene.

The *mucin 1* (*MUC1*) gene, which first appeared in mammals, is expressed at the apical borders of polarized epithelial cells exposed to the external environment^[3,4]^. *MUC1* encodes a single polypeptide that undergoes auto-cleavage into N-terminal (MUC1-N) and C-terminal (MUC1-C) subunits^[5]^. MUC1-N and MUC1-C form a non-covalent complex that is positioned at the apical cell membrane [Figure 1]^[5]^. The extracellular MUC1-N subunit includes variable numbers of 60 bp tandem repeats that are highly glycosylated, resulting in a structure that extends beyond the glycocalyx and contributes to a protective mucous barrier [Figure 1]^[5]^. The MUC1-C subunit includes extracellular, transmembrane, and cytoplasmic domains that function in the activation of inflammatory, proliferative, and repair pathways associated with the wound healing response [Figure 1]^[6]^.

MUC1-N/MUC1-C heterodimers at the epithelial cell membrane, which act as sensors of entropic forces within the extracellular matrix, are disrupted by the loss of homeostasis^[6–8]^. As a result, MUC1-N is shed into the protective physical barrier, and MUC1-C becomes available for transducing stress signals from the cell membrane to the interior of the epithelial cell^[6]^[Figure 1]. MUC1-C activates the epithelial-mesenchymal transition (EMT) with loss of apical-basal polarity^[9]^. In this way, MUC1-C is expressed over the entire cell membrane, where it contributes to the activation of receptor tyrosine kinases (RTKs) and their downstream signaling pathways, linking inflammation and proliferation^[5,6]^. Of importance, MUC1-C undergoes endocytic internalization from the cell membrane^[10]^ and is imported into the nucleus by interacting with the cytoplasmic face of the nuclear pore complex [Figure 1]^[6,11]^.

MUC1 was identified by its overexpression in breast and other human cancers^[3]^. These findings and involvement of the MUC1-C subunit in driving hallmarks of the cancer cell, including the cancer stem cell (CSC) state and capacity for self-renewal, have supported the notion that chronic activation of MUC1-C plays a role in promoting the progression of the resident SC wound healing response to cancer^[13]^. Recent evidence, which lends support to that premise and the proposal by Rudolf Virchow that “cancer is a wound that does not heal”^[14]^, is presented in this review.

## MUC1-C DRIVES LINEAGE PLASTICITY AND EPIGENETIC REMODELING IN CANCER PROGRESSION

**Figure 2.**
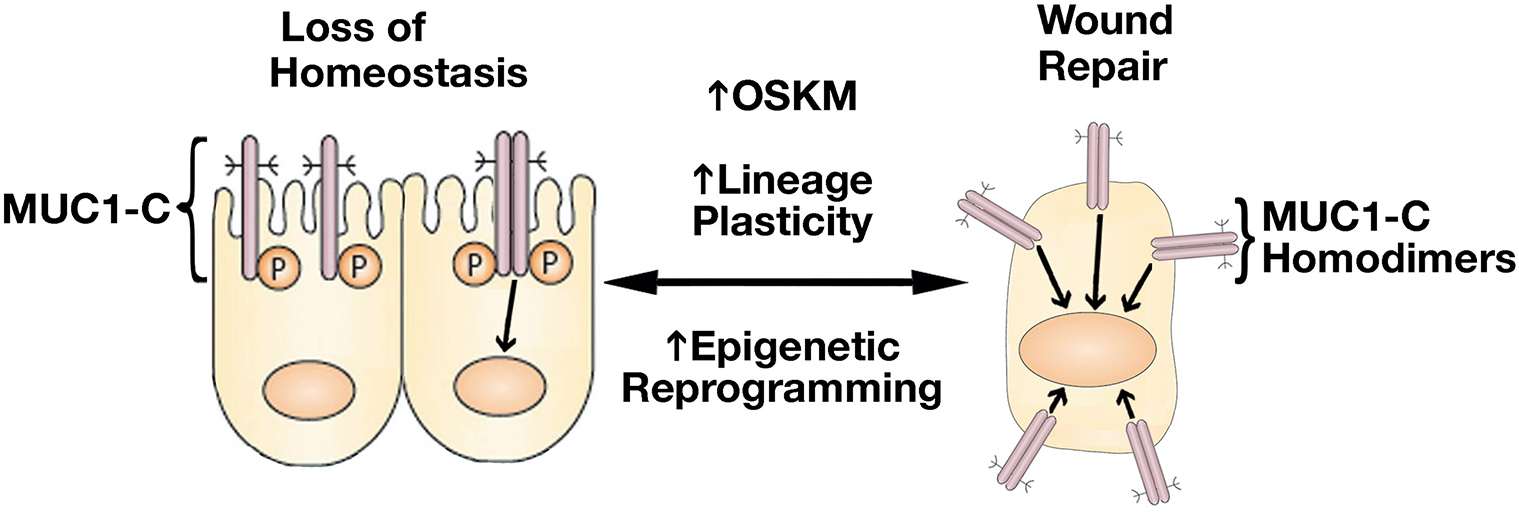
Activation of MUC1-C in response to the loss of homeostasis. Activation of MUC1-C induces homodimers mediated by a redox-sensitive CQC motif in the cytoplasmic domain^[22]^. MUC1-C homodimers are imported into the nucleus where they interact with transcription factors, such as STAT3, NF-κB, MYC, and E2F, that induce (i) the Yamanaka OSKM pluripotency factors, (ii) lineage plasticity with EMT, and (iii) effectors of epigenetic reprogramming^[23,24]^. In this way, MUC1-C reversibly couples pluripotency and lineage plasticity with epigenetic reprogramming that govern wound repair.

Resident SC lineage infidelity is transient in the wound healing response and, in contrast, is persistent in cancer^[2]^, albeit by presently unclear mechanisms that conceivably involve epigenetic memory. MUC1-C regulates lineage plasticity in driving progression to the highly aggressive form of neuroendocrine prostate cancer (NEPC)^[15]^. MUC1-C is overexpressed in castration-resistant prostate cancer (CRPC) and NEPC, and induces the Yamanaka OCT4, SOX2, KLF4, and MYC (OSKM) pluripotency factors^[15]^ [Figure 2]. Seminal work by Yamanaka showed that the OSKM factors are sufficient to induce somatic cells to pluripotent stem cells (iPSCs)^[16,17]^. iPSCs and CSCs share gene signatures and other characteristics associated with lineage plasticity^[18–20]^. In somatic cells, pluripotency factor expression is restricted to maintain lineage specification, whereas these factors are activated transiently in wound healing and persistently in cancer progression^[21]^. The finding that MUC1-C induces the Yamanaka pluripotency factors in CRPC→NEPC progression thus provided new insights into the chronic induction of OSKM expression and lineage plasticity in cancer^[15]^ [Figure 2].

The epithelial wound response includes phases of inflammation, proliferation, and remodeling that are resolved with healing^[25–27]^. The highly prevalent emergence of chronic inflammation has been promoted by changes in societal, environmental, and dietary factors that contribute to cancer^[28]^. Along these lines, chronic inflammation with prolonged cycles of damage and repair is an established driver of cancer initiation and progression^[29]^. Studies in a colitis model of chronic inflammation demonstrated upregulation of MUC1-C and the OSM+NANOG pluripotency factors^[13]^. Targeting MUC1-C suppressed OSM+NANOG expression and the progression of colitis to colon carcinoma, supporting the involvement of MUC1-C in resident SC lineage plasticity in a setting of chronic inflammation^[13]^. The involvement of MUC1-C in driving the Yamanaka pluripotency factors and lineage plasticity are conceivably conferred by chronic inflammation in the progression of other types of cancers. In support of this notion, MUC1-C induces the Yamanaka factors and lineage infidelity in CRPC→NEPC progression^[15]^, dedifferentiation of triple-negative breast cancer (TNBC)^[30]^, and pancreatic ductal carcinomas with NE features^[31]^.

### MUC1-C merges lineage plasticity with DNA and histone reprogramming

**Figure 3.**
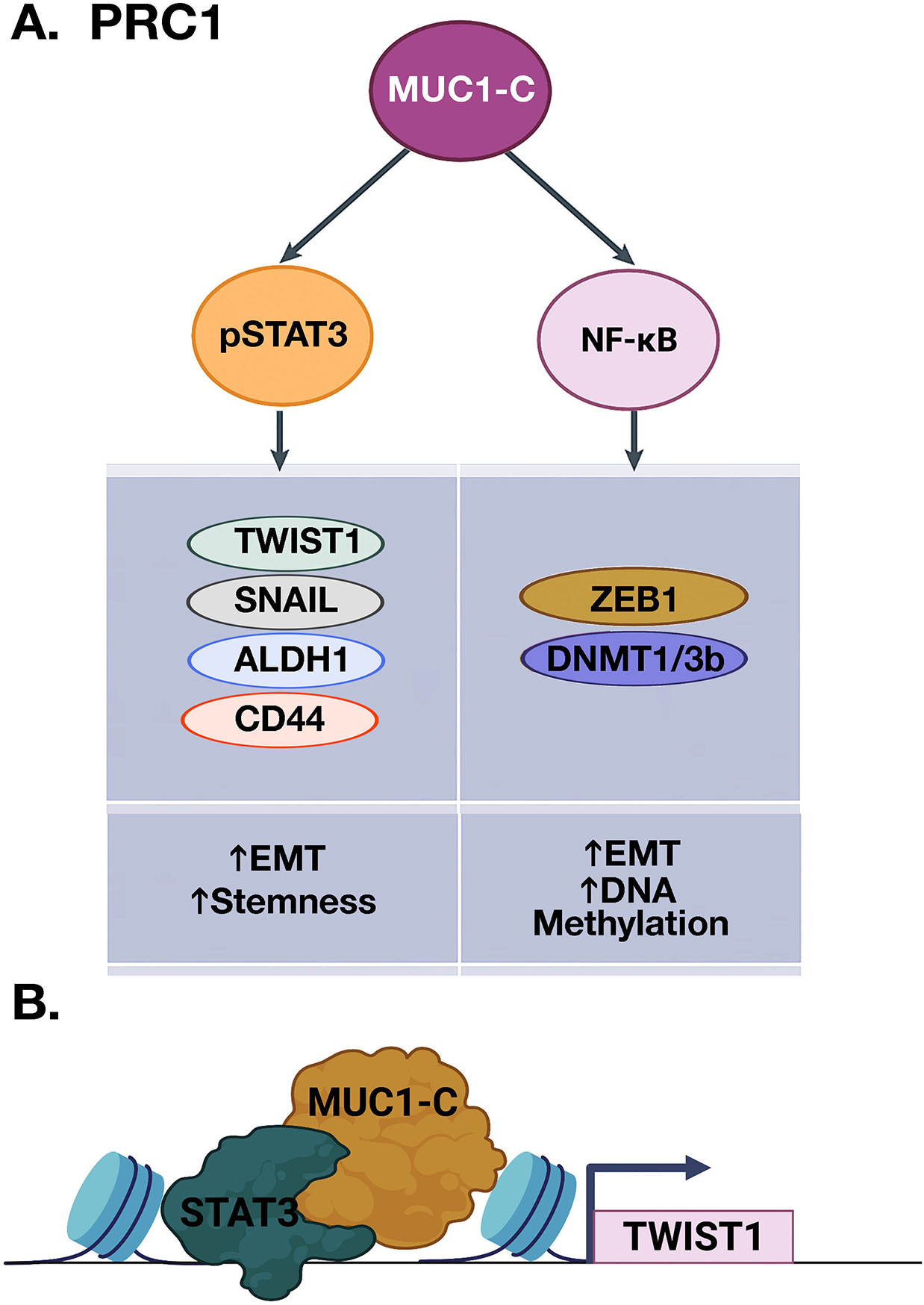
MUC1-C activates inflammatory pSTAT3 and NF-κB signaling in integrating EMT and stemness with DNA methylation. A. MUC1-C promotes STAT3 phosphorylation and forms a complex with pSTAT3 that induces expression of TWIST1, SNAIL, ALDH1, and CD44 (left). MUC1 also binds directly to NF-κB, promotes occupancy of NF-κB on the ZEB1 and DNMT1/3b promoters, and induces their expression (right). In turn, MUC1-C forms a complex with ZEB1 that recruits DNMTs, induces DNA methylation of the *CDH1* CpG island, and suppresses CDH1 expression. B. MUC1-C interacts with TFs, such as pSTAT3 and, while not binding directly to DNA, forms complexes on target genes to promote their activation, as shown for *TWIST1* (BioRender), as well as repression of others^[23,24]^.

Lineage plasticity of cells that have acquired migratory characteristics of the EMT program contributes to re-epithelialization in the wound healing response^[26,27]^. EMT is driven by the activation of EMT-TFs, such as TWIST1, SNAIL, and ZEB1^[32,33]^. MUC1-C activates the inflammatory STAT3 and NF-κB p65 pathways in auto-inductive loops that drive upregulation of MUC1 expression^[34–36]^. In this way, MUC1-C induces JAK1-mediated pSTAT3 activation^[36]^. In turn, MUC1-C forms complexes with pSTAT3 that induce TWIST1 expression [Figure 3A and 3B]^[30,36]^. MUC1-C binds directly to TWIST1 and induces SNAIL and stemness-associated factors, such as ALDH1 and CD44^[6,30,37]^. MUC1-C→NF-κB signaling also induces the *ZEB1* gene and MUC1-C forms a direct complex of the ZEB1 protein^[9]^. As a result, MUC1-C promotes the repression of ZEB1 target genes, such as miR-200, a suppressor of EMT and CRB3, coupling induction of EMT with disruption of the CRUMBS polarity complex^[9,38]^.

**Figure 4.**
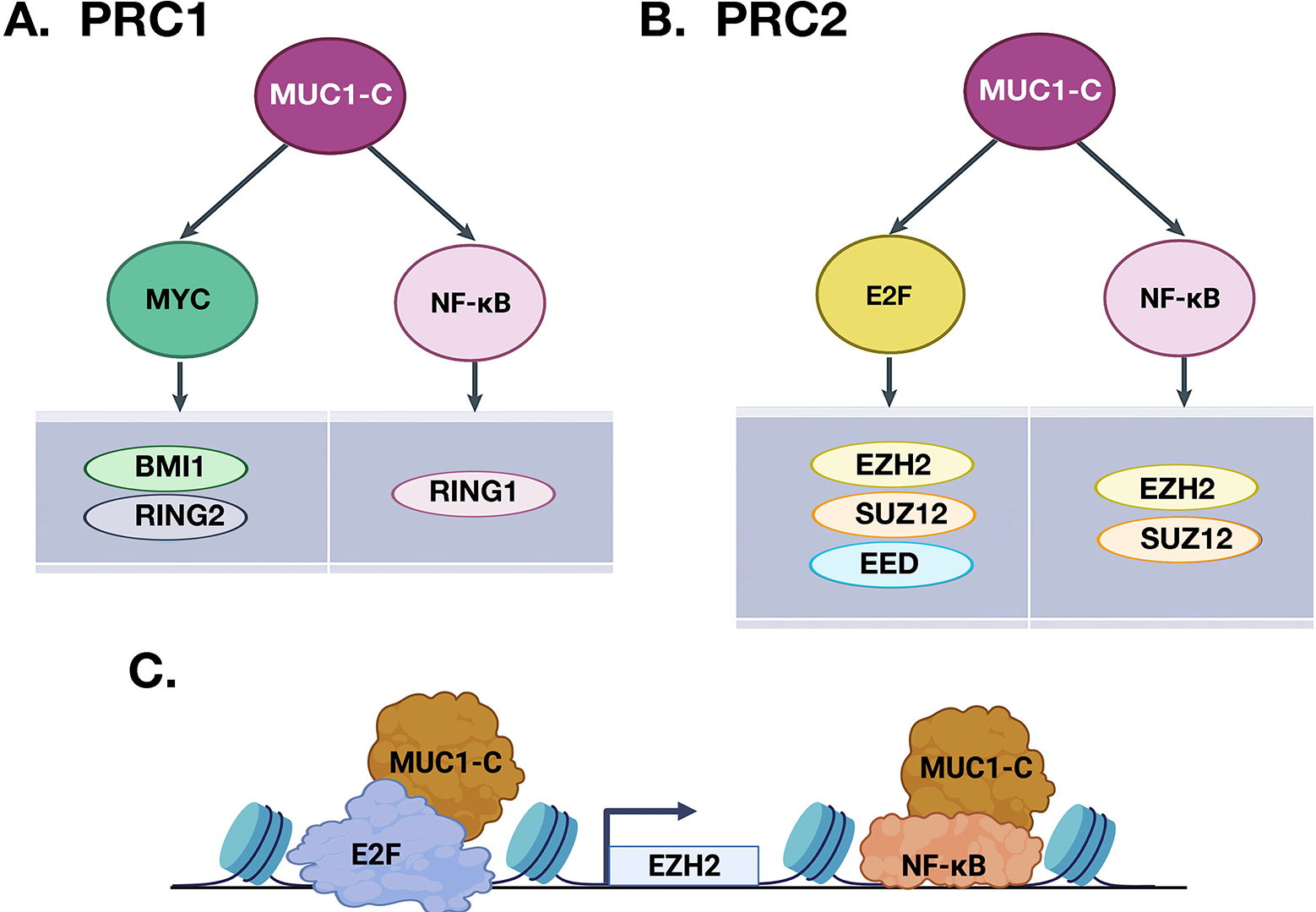
MUC1-C couples activation of NF-κB, MYC, and E2F in inducing the repressive PRC1 and PRC2 complexes. MUC1-C forms distinct complexes with NF-κB, MYC, and E2F1. A. MUC1-C/MYC and MUC1-C/NF-κB complexes activate PRC1 by inducing BMI1, RING2, and RING1. B. The MUC1-C→E2F and MUC1-C→NF-κB pathways activate PRC2 by inducing EZH2, SUZ12, and EED. C. Schema depicting the integration of MUC1-C-induced activation of E2F and NF-κB in driving EZH2 expression (BioRender). These pathways and those shown in Figures 3 and 5 are highlighted to emphasize the capacity of MUC1-C to integrate the activation of multiple effectors that reprogram the epigenome. The formation of specific MUC1-C complexes on the promoters and enhancers of target genes has also been summarized elsewhere^[23,24]^.

Lineage plasticity and EMT are intricately linked to reprogramming of the epigenome^[39–41]^. The MUC1-C→NF-κB pathway, which induces ZEB1 and EMT, activates DNA methyltransferase 1 (DNMT1) and DNMT3b with hypermethylation of the *CDH1* promoter and downregulation of E-cadherin expression^[42]^. The MUC1-C→NF-κB pathway also induces expression of the RING1 protein that is responsible for the catalytic activity of the Polycomb Repressive Complex 1 (PRC1)^[43]^. In activating the PRC1 complex, MUC1-C intersects induction of inflammatory NF-κB signaling with the MYC pathway [Figure 4A]^[24]^. MUC1-C induces *MYC* expression by directly activating the WNT/β-catenin/TCF4 pathway^[44–47]^. Consistent with inducing TF-encoding genes and then forming complexes with their products to promote occupancy of target genes, MUC1-C binds to the MYC HLH-LZ domain and regulates the MYC transactivation function^[30]^. In this way, MUC1-C→MYC signaling activates the expression of BMI1 and RING2, which, together with RING1, constitute the PRC1 complex [Figure 4A]^[24, 43]^.

MUC1-C→MYC signaling also activates the NuRD chromatin remodeling and deacetylation complex^[30]^, which is recruited by ZEB1 and TWIST EMT-TFs to their target genes, such as *CDH1*, and facilitates PRC1-mediated gene repression^[40,48,49]^. NuRD positions nucleosomes at regulatory DNA elements, removes activating H3K27 acetylation marks, and facilitates recruitment of PRC1^[49]^. NuRD consists of core chromodomain-helicase-DNA-binding proteins CHD3 and CHD4 that catalyze ATP-dependent chromatin remodeling in association with the histone deacetylase HDAC1 and HDAC2 subunits^[48]^. NuRD also includes the non-enzymatic (i) methyl-CpG binding domain MBD2 and MBD3, (ii) metastasis-associated gene 1 (MTA1), and (iii) retinoblastoma-binding RBBP4 and RBBP7 proteins^[48]^. The MUC1-C→MYC pathway induces *MTA1* and *MBD3* expression and post-transcriptionally upregulates CHD4 levels^[30]^. MUC1-C also forms a nuclear complex with NuRD in association with promoting the CSC state^[30]^.

Chromatin occupancy of canonical PRC1 complexes is dependent on binding to Polycomb Repressive Complex 2 (PRC2)-deposited H3K27me3^[50]^. PRC2 consists in part of the EZH2, SUZ12, and EED subunits^[51]^. In contrast to the regulation of PRC1 by NF-κB and MYC, MUC1-C consolidates the NF-κB and E2F pathways in activating PRC2 [Figure 4B]^[24,51]^. As shown for NF-κB^[35]^, MUC1-C binds directly to E2F1 and promotes the induction of E2F1 target genes^[52]^. MUC1-C induces *EZH2* and *SUZ12* by NF-κB-mediated activation of their enhancer regions^[24,51]^. In addition, MUC1-C induces *EZH2* and *SUZ12* by E2F-driven activation of their promoter regions [Figure 4B]^[24,51]^. MUC1-C also induces EED by E2F, but not NF-κB, signaling^[24,51]^. Of importance for integrating these pathways, MUC1-C induces *EZH2* expression by E2F- and NF-κB-mediated activation of the *EZH2* promoter and enhancer regions, respectively [Figure 4C].

In summary, the MUC1-C→NF-κB pathway consolidates lineage plasticity with activation of (i) ZEB1 and EMT, and (ii) DNMT1/3b and DNA methylation. The MUC1-C→NF-κB and MUC1-C→MYC pathways activate PRC1, whereas MUC1-C→MYC signaling induces NuRD. Furthermore, the MUC1-C→NF-κB and MUC1-C→E2F1 pathways activate PRC2. These findings indicate that MUC1-C integrates inflammatory NF-κB, proliferative E2F, and remodeling MYC pathways in reprogramming DNA and histones by DNMT1/3b, PRC1/2, and NuRD.

### MUC1-C regulates the intersection of the Polycomb and Trithorax families of epigenetic remodelers

**Figure 5.**
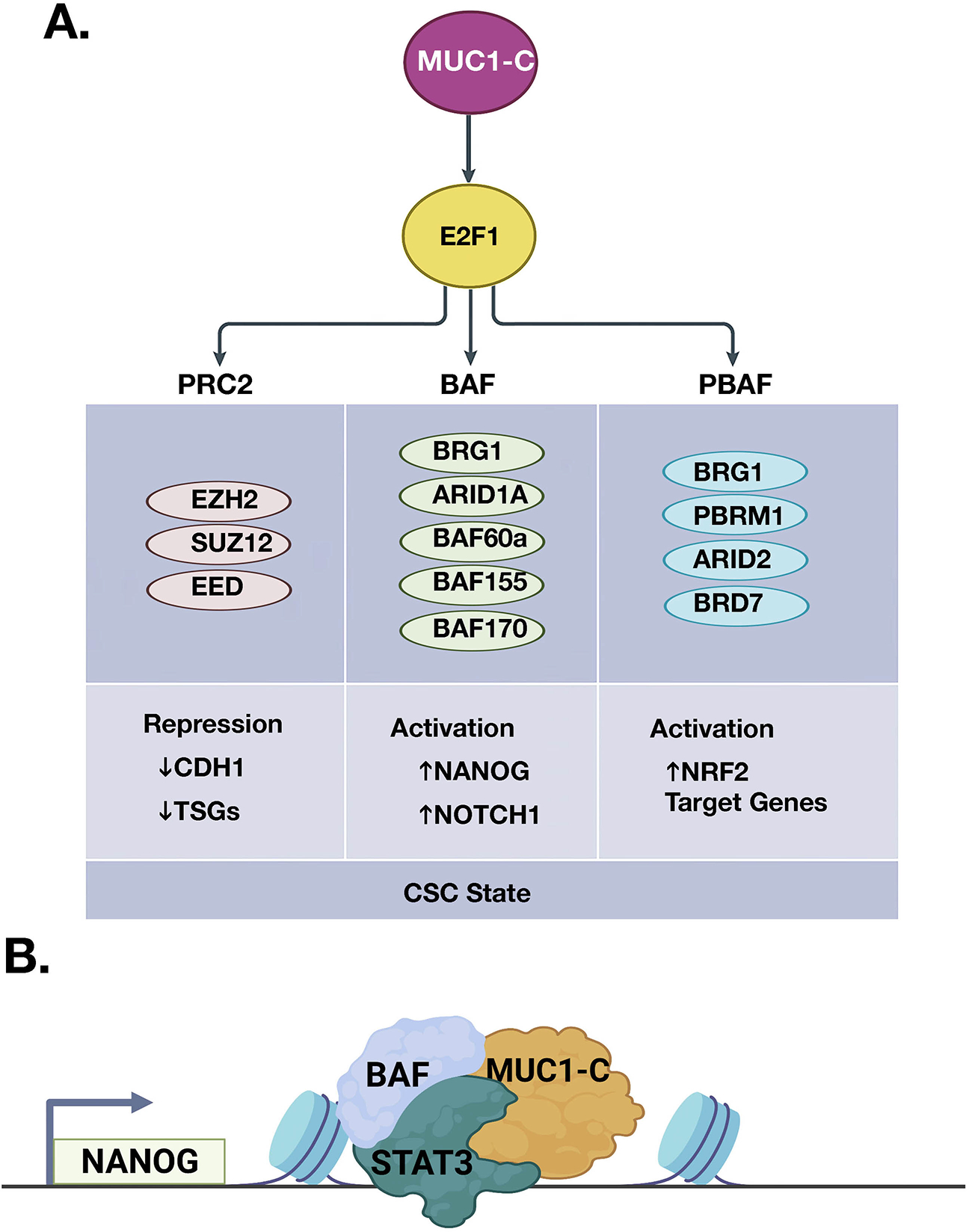
MUC1-C→E2F1 pathway intersects induction of PcG and TrxG complexes. A. The PcG/PRC2 components EZH2, SUZ12, and EED are activated by MUC1-C/E2F complexes. The binding of MUC1-C to EZH2 induces H3K27 trimethylation with the repression of *CDH1* and other TSGs. MUC1-C→E2F1 signaling also activates the TrxG SWI/SNF (BAF, PBAF) chromatin remodeling complexes. MUC1-C associates with nuclear (i) BAF in inducing the NANOG pluripotency factor and the NOTCH1 stemness gene, and (ii) PBAF in activating NRF2 target genes that regulate redox balance. In doing so, MUC1-C→E2F1 signaling has the capacity to fine-tune the intersection of PcG and TrxG proteins in driving the CSC state. B. Formation of MUC1-C complexes with BAF and PBAF on specific target genes, such as *NANOG* (BioRender), has been detailed in recent publications^[52,56,57]^. CSC: Cancer stem cell.

The PRC1/2 Polycomb group (PcG) proteins repress gene expression by BMI1-driven H2A K119 ubiquitylation and EZH2-mediated H3K27 methylation^[53]^. In addition to inducing BMI1 expression, MUC1-C binds directly to BMI1 and thereby enhances H2AK119 ubiquitylation and repression of the (i) *CDKN2A* TSG and (ii) *HOXC5* and *HOXC13* homeotic genes^[43]^. MUC1-C induces EZH2 expression and binds directly to EZH2 at the CXC domain adjacent to the catalytic SET region^[51]^. As a result, MUC1-C increases EZH2 occupancy on TSGs, such as *CDH1* and *BRCA1*, and drives H3K27 trimethylation and repression [Figure 5]^[51]^. This pattern of inducing PcG expression and interacting with the PcG protein product is analogous to that observed with TFs, such as ZEB1^[9]^, TWIST1^[54]^, and MYC^[30,46,47]^, among others. Why this dual regulation of PcG proteins and TFs occurs is not clear; however, one explanation could be that, in wound healing, MUC1-C rapidly interacts with existing pools of these effectors and then induces their expression to sustain activation of the repair process. By extension, constitutive MUC1-C activation driven by STAT3- and NF-κB-mediated auto-inductive circuits in cancer cells could promote established inflammatory memory with irreversible transcriptional and post-transcriptional induction of these pathways^[6,55]^.

PcG-induced gene repression is counteracted by the Trithorax group (TrxG) of SWI/SNF chromatin remodelers and COMPASS family of histone lysine methyltransferases that promote transcriptional activation^[53]^. The balance of PcG and TrxG proteins in regulating gene expression is critical for normal development and, when disrupted, contributes to cancer progression^[53]^. Studies in models of CRPC and TNBC CSCs showed that, in addition to PRC2, MUC1-C→E2F1 signaling induces BRG1, ARID1A, and other components of the SWI/SNF BAF complex [Figure 5A]^[52]^. MUC1-C associates with nuclear BAF, regulates the core pluripotency network, and induces CSC gene signatures. In this way, the MUC1-C→E2F1→BAF pathway induces the (i) NANOG pluripotency factor, (ii) NOTCH1 effector of the CSC state, and (iii) capacity for self-renewal [Figure 5A and 5B]^[52]^. The SWI/SNF polybromo-associated BAF (PBAF) complex also includes BRG1, as well as the distinct PBRM1, ARID2, and BRD7 subunits^[58]^. PBAF regulates cell differentiation, genomic integrity, and redox balance^[58–60]^. As found for BAF, MUC1-C activates *PBRM1*, *ARID2*, and *BRD7* by E2F1-mediated signaling^[56]^. MUC1-C is also associated with nuclear PBAF and the NRF2 TF in inducing the expression of genes, such as *SLC7A11*, *G6PD,* and *PDG*, that encode effectors of redox balance^[56]^. Additionally, MUC1-C merges the BAF and PBAF pathways in promoting lineage plasticity and the CSC state^[56]^.

In summary, MUC1-C→E2F signaling induces (i) EZH2 and H3K27me3 in gene repression, and (ii) BRG1, which removes PRC2-mediated repressive marks through chromatin remodeling. The MUC1-C→E2F pathway thus has the capacity to control the balance of PcG and TrxG complexes in regulating gene expression in wound repair, as well as their dysregulation in cancer. The COMPASS family replaces PcG-driven repressive marks by inducing H3K4 methylation and transcriptional activation^[53]^. Emerging evidence indicates that MUC1-C plays a role in driving H3K4 trimethylation of enhancer-like signatures of stemness-associated genes^[57]^. Additional studies will therefore be needed to determine whether MUC1-C activates COMPASS and thereby the H3K4me3 mark on these target genes.

### MUC1-C regulates chromatin accessibility of enhancers in CSC models

Enhancers function as binding platforms for integrating the action of somatic TFs, such as the JUN/AP-1 family that regulate proliferation and differentiation^[61]^. The Yamanaka OSK factors drive the reorganization of enhancers during reprogramming, whereas MYC predominantly acts at promoters^[62]^. In addition, the OSK factors indirectly silence somatic enhancers and open chromatin by direct sequence-specific binding to DNA in activating pluripotency enhancers^[62]^. Direct OSK-mediated reprogramming of enhancers is dependent on the recruitment of the BAF chromatin remodeling complex^[63–65]^. As a consequence, the BRG1 ATPase subunit of the BAF complex continuously maintains enhancers with open chromatin for binding of OSK and promotes the removal of flanking nucleosomes for occupancy of additional TFs^[66,67]^. BRG1 also interacts directly with the transcription factor CCCTC-binding factor (CTCF), which plays a role in largescale genome organization^[68,69]^.

**Figure 6.**
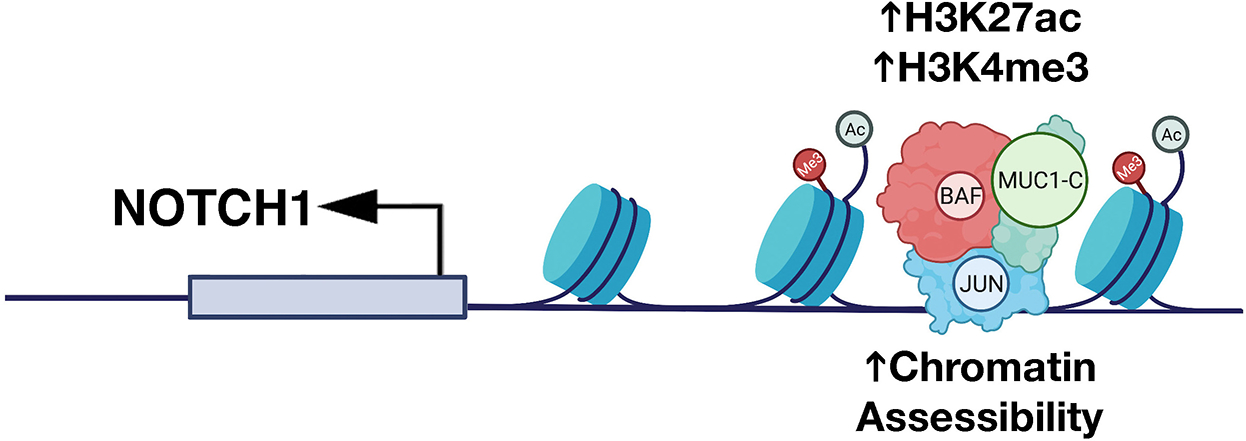
MUC1-C dictates JUN and BAF-mediated chromatin remodeling at a proximal enhancer-like signature (pELS) in the NOTCH1 gene. MUC1-C interacts directly with JUN to form a complex that occupies a pELS in the NOTCH1 gene. In turn, MUC1-C-JUN complexes recruit BAF, as evidenced by MUC1-C-dependent occupancy of both JUN and ARID1A. MUC1-C is necessary for increasing (i) chromatin accessibility, and (ii) H3K27ac and H3K4me3 marks at the pELS in association with the activation of NOTCH1 expression. Modified from Bhattacharya^[57]^.

The findings that MUC1-C activates expression of the OSK factors and the BAF complex in cancer cells invoked the possibility that MUC1-C regulates chromatin accessibility of enhancers in promoting the CSC state. In support of this notion, ATAC-seq studies in models of MUC1-C-induced CRPC and TNBC progression showed that MUC1-C drives global changes in chromatin architecture^[57]^. MUC1-C induces differentially accessible regions (DARs) across the CRPC and TNBC genomes, which are significantly associated with differentially expressed genes (DEGs)^[57]^. Motif and cistrome analysis further demonstrated that MUC1-C-induced DARs align with genes regulated by the JUN/AP-1 family. As identified for OSK pluripotency factors^[62]^, JUN recruits BAF in enhancer selection^[70]^. In studies of the *NOTCH1* stemness gene, MUC1-C was shown to be necessary for (i) occupancy of JUN and ARID1A/BAF, (ii) increases in H3K27ac and H3K4me3 signals, and (iii) opening of chromatin on a proximal enhancer-like signature [Figure 6]. Studies of the *EGR1* stemness-associated gene and the *LY6E* gene, which has been linked to both stemness and immune evasion, further demonstrated that MUC1-C-induced JUN/ARID1A complexes regulate chromatin accessibility on proximal and distal enhancer-like signatures^[57]^. These findings uncovered a role for MUC1-C in chromatin remodeling that is mediated at least in part by induction of pluripotency factors, JUN/AP-1 and BAF, in association with driving the CSC state. Further studies will now be needed to determine if MUC1-C also plays a role in (i) recruitment of the PBAF complex in the inflammatory memory response, and (ii) regulation of CTCF and topologically associated domains (TADs).

## SUMMARY

**Figure 7.**
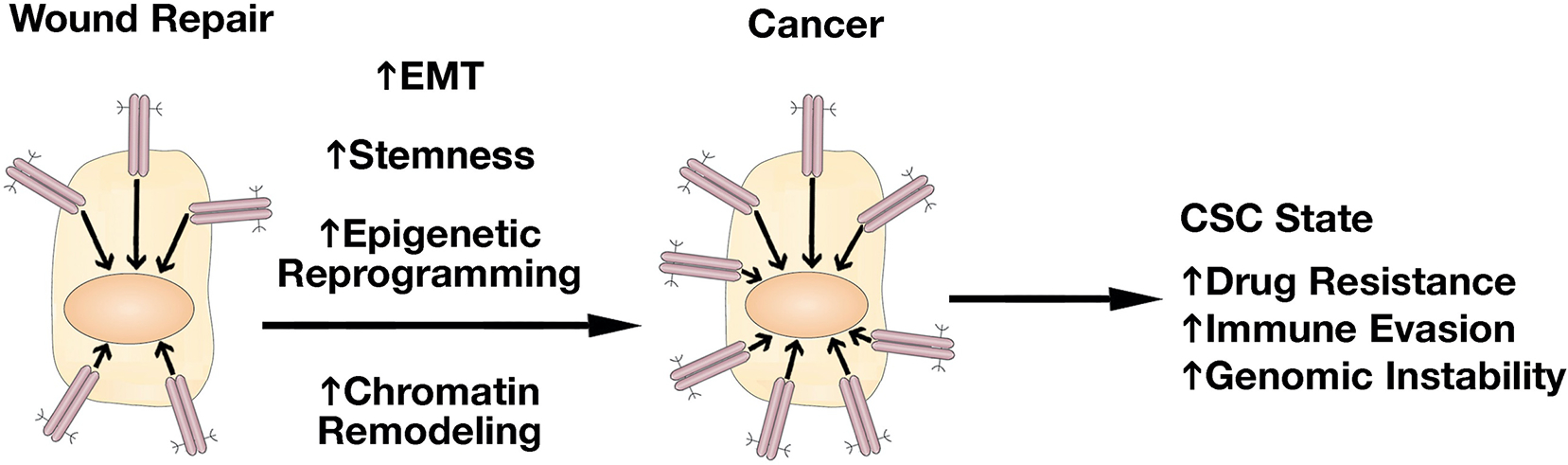
MUC1-C drives the progression of wound repair to cancer. In response to the loss of homeostasis, MUC1-C activates inflammatory STAT3 and NF-κB pathways that induce EMT, stemness, and methylation of DNA and histones. These pathways are integrated with activation of E2F and MYC in driving reversible proliferative and chromatin remodeling pathways associated with the resident SC inflammatory memory response. In settings of chronic inflammation, prolonged MUC1-C activation irreversibly establishes this memory response and drives the CSC state with drug resistance, immune evasion, genomic instability, and poor clinical outcomes. EMT: Epithelial-mesenchymal transition; SC: stem cells; CSC: cancer stem cell.

The field of MUC1-C research in cancer progression has been advanced by uncovering the involvement of MUC1-C in integrating induction of lineage plasticity, DNA and histone modifications and more recently changes in chromatin accessibility with the CSC state [Figure 7]. Dedifferentiation and lineage plasticity endows cancer cells with the capacity for drug resistance and for evasion of immune recognition and destruction^[71–74]^. Along these lines, MUC1-C drives the resistance of cancer cells to cytotoxic and targeted agents^[6]^. MUC1-C also induces signaling pathways that promote immune evasion and immune cell-depleted “cold” TMEs that associate with resistance to immune checkpoint inhibitors^[6,75,76]^. As a result, MUC1-C has emerged as a highly attractive target for the development of direct inhibitors, as well as immune-based approaches, such as CAR T cells and antibody-drug conjugates^[6]^.

Of importance, chronic activation of MUC1-C is not associated with specific intrinsic mutations as commonly occurs with oncogenes; rather, MUC1-C-induced oncogenesis is driven by inflammatory and epigenetic remodeling pathways that, when persistent, establishes an irreversible resident SC memory response and promotes cancer progression [Figure 7]. Nonetheless, MUC1-C-driven genomic instability could promote the accumulation of mutations in MUC1-C-induced downstream effectors, such as BAF and others, that contribute to the CSC state [Figure 7]^[6]^. In further substantiating this notion, studies will be needed that address, at least in part, how MUC1-C-induced epigenetic reprogramming in wound repair, which is transient and associated with inflammatory memory, becomes sustained and irreversible in driving oncogenesis.
